# Pleuropulmonary Blastoma Presenting as an Oncologic Emergency

**DOI:** 10.7759/cureus.103342

**Published:** 2026-02-10

**Authors:** Ryo Tamura, Shohei Yoshimura, Kotaro Uemura, Chieko Hisamatsu, Natsuko Yoshikawa, Suguru Uemura, Hiroshi Kurosawa, Makiko Yoshida, Daiichiro Hasegawa, Tadashi Hatakeyama

**Affiliations:** 1 Department of Pediatric Surgery, Hyogo Prefectural Kobe Children's Hospital, Kobe, JPN; 2 Department of Pediatric Surgery, Japanese Red Cross Society Himeji Hospital, Himeji, JPN; 3 Department of Hematology/Oncology, Hyogo Prefectural Kobe Children's Hospital, Kobe, JPN; 4 Center for Pediatric Critical Care Medicine, Hyogo Prefectural Kobe Children's Hospital, Kobe, JPN; 5 Department of Pathology, Hyogo Prefectural Kobe Children's Hospital, Kobe, JPN

**Keywords:** emergency thoracotomy, oncologic emergencies, pediatric oncology treatment, pediatric onco-surgery, pleuropulmonary blastoma (ppb)

## Abstract

Oncologic emergency (OE) is a life-threatening condition caused by a tumor or its treatment and may result from severe mass effect on vital organs. Although thoracic OE are most commonly associated with anterior mediastinal tumors, large intrathoracic tumors can also cause critical cardiopulmonary compromise. We report a rare case of pleuropulmonary blastoma (PPB) presenting as a thoracic OE. A three-year-old girl was transferred with severe respiratory distress due to complete right lung collapse and tracheal deviation to the left because of a large intrathoracic tumor. Pre-emptive intubation to secure the airway was avoided due to a concern for respiratory decompensation. However, further investigation, including percutaneous biopsy and contrast-enhanced computed tomography (CT), required sedation and endotracheal intubation, which subsequently led to deterioration of cardiopulmonary function. Because urgent chemotherapy for the reduction of tumor size was not feasible, emergency surgical decompression was performed. A hemi-clamshell thoracotomy allowed prompt exteriorization of the tumor, decompression of the thoracic cavity, and safe right lower lobectomy. Histopathology confirmed pleuropulmonary blastoma type II. The postoperative course was uneventful, and the patient remains disease-free after adjuvant chemotherapy. This case highlights that pleuropulmonary blastoma can present as a fulminant OE and demonstrates that early surgical decompression using a hemi-clamshell approach can be lifesaving in rapidly deteriorating patients.

## Introduction

Oncologic emergency (OE) is a life-threatening condition caused either by the tumor itself or by its treatment [1]. The etiology varies according to the tumor type and its anatomical location. In the thorax, airway obstruction and cardiopulmonary compromise are the most critical manifestations of OE and are most commonly reported in anterior mediastinal tumors because of their close proximity to vital structures [2]. However, similar catastrophic conditions can be caused by large intrathoracic tumors, although such presentations are seldom reported [3]. Pleuropulmonary blastoma (PPB) is the most common pulmonary malignancy in childhood, with a prevalence of around one and one and a half million children per year [4]; however, it accounts for only 0.25%-0.5% of all pulmonary malignancies across all age groups [5]. Its clinical presentation is highly variable, ranging from asymptomatic incidental findings to severe cardiopulmonary compromise [6]. Herein, we report a case of pleuropulmonary blastoma (PPB) presenting as an OE that was promptly managed with an excellent outcome.

## Case presentation

**Figure 1:**
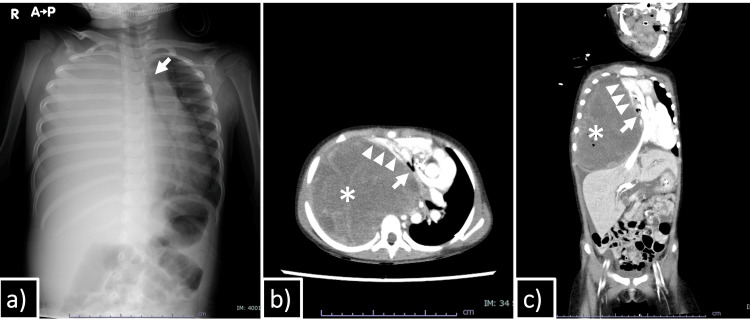
Chest X-ray and contrast-enhanced CT Chest radiograph obtained on the day of referral showed complete opacification of the right hemithorax and deviation of the trachea (arrow) (a). Axial (b) and coronal (c) contrast-enhanced CT images demonstrated a large tumor (asterisk) occupying the entire right thoracic cavity, with the normal lung parenchyma (arrowheads) displaced anteromedially. The trachea (arrow) was deviated and markedly narrowed. CT: computed tomography

A three-year-old girl was transferred from a local hospital because of severe dyspnea and complete collapse of the right lung on chest radiography (Figure 1a). Two weeks prior to admission, she was brought to a local clinic for fever and dyspnea and was treated as having an acute asthma attack; however, her dyspnea did not improve. Therefore, she received chest radiography that revealed complete right lung collapse. Her past medical history was unremarkable, except for resection of hamartomas of the gingiva and tongue at one year of age. There was no history of weight loss.

On admission, she was alert and afebrile but in significant respiratory distress. Her respiratory rate was 30 breaths per minute, and she required 6 L/minute of supplemental oxygen to maintain peripheral oxygen saturation (SpO₂) above 90%. Marked chest wall retractions were observed during respiration. Breath sounds were absent over the right hemithorax, while normal breath sounds without wheezing, rhonchi, or crackles were noted on the left. Non-contrast-enhanced computed tomography (CT) demonstrated complete obliteration of the right thoracic cavity by a mass, with the residual lung parenchyma displaced anteromedially. The patient was admitted to the pediatric intensive care unit. A multidisciplinary conference involving pediatric intensive care doctors, anesthetists, cardiovascular surgeons, and pediatric surgeons was held to discuss pre-emptive intubation for securing the airway; however, the procedure was postponed because of concern that even mild sedation could further compromise her respiratory status. Nevertheless, the patient was too agitated to tolerate percutaneous needle biopsy or contrast-enhanced CT under minimal sedation. Therefore, endotracheal intubation was ultimately performed with cardiovascular surgeons on standby for emergent extracorporeal membrane oxygenation (ECMO). As anticipated, high airway pressures and positive end-expiratory pressure (PEEP) were required to maintain airway patency under sedation, although cardiopulmonary stability was preserved during diagnostic evaluation. Contrast-enhanced CT revealed a large, heterogeneously enhancing tumor occupying the entire right hemithorax, causing complete collapse of the right lung and leftward mediastinal shift, suggesting origin from the right lower lobe. A small amount of pleural effusion was also present (Figure 1b, 1c). Ultrasound-guided fine needle aspiration biopsy demonstrated polymorphic cellular proliferation with atypical nuclei and eosinophilic cytoplasm, findings consistent with pleuropulmonary blastoma. Given the diagnosis of PPB presenting as an OE, urgent chemotherapy was considered to reduce tumor size. However, the patient's cardiopulmonary status progressively deteriorated, and metabolic acidosis worsened, necessitating high PEEP and pressure support, as well as repeated fluid boluses and inotropic support. Ultimately, emergency thoracotomy for decompression of the chest cavity and mediastinum was decided one day after intubation.

**Figure 2:**
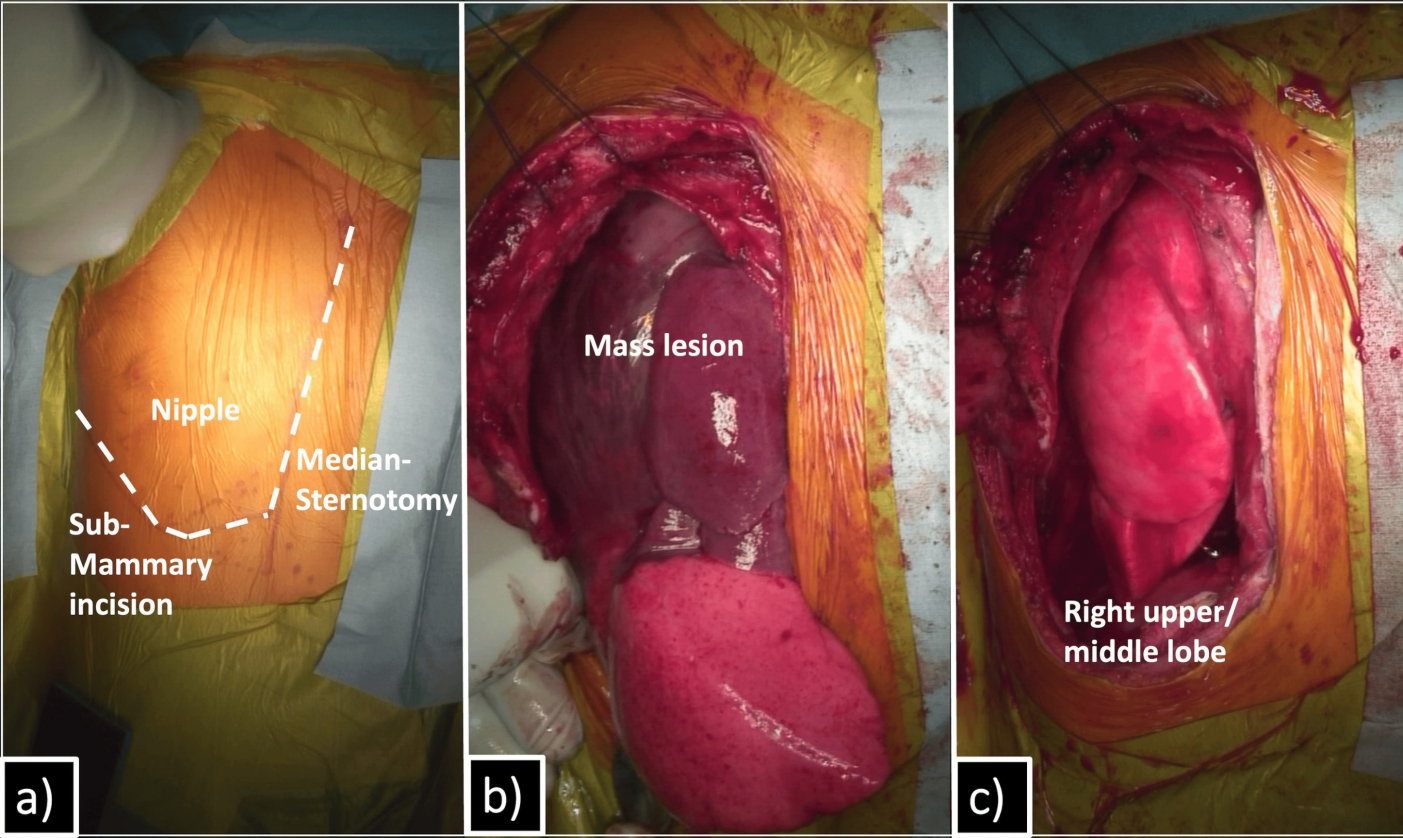
Surgical findings A hemi-clamshell incision was performed (a). The tumor was exteriorized through the thoracotomy and was found to originate from the right lower lobe (b). Right lower lobectomy was subsequently performed (c).

A hemi-clamshell thoracotomy was employed to achieve complete exposure of the large tumor. It emerged from the right lower lobe, with multiple metastatic lesions on both the visceral and parietal pleura (Figure 2a-2c). Although there was no severe adhesion to the mediastinum or anterior chest wall, the tumor was firmly adherent to the posterior chest wall. Right lower lobectomy with hilar lymph node sampling was performed along with the removal of metastatic lesions with surrounding parietal pleura. Complete pleurectomy was not performed, but gross removal of the entire metastatic lesions was achieved. Tumor capsule rupture occurred posteriorly because of dense adhesions.

**Figure 3:**
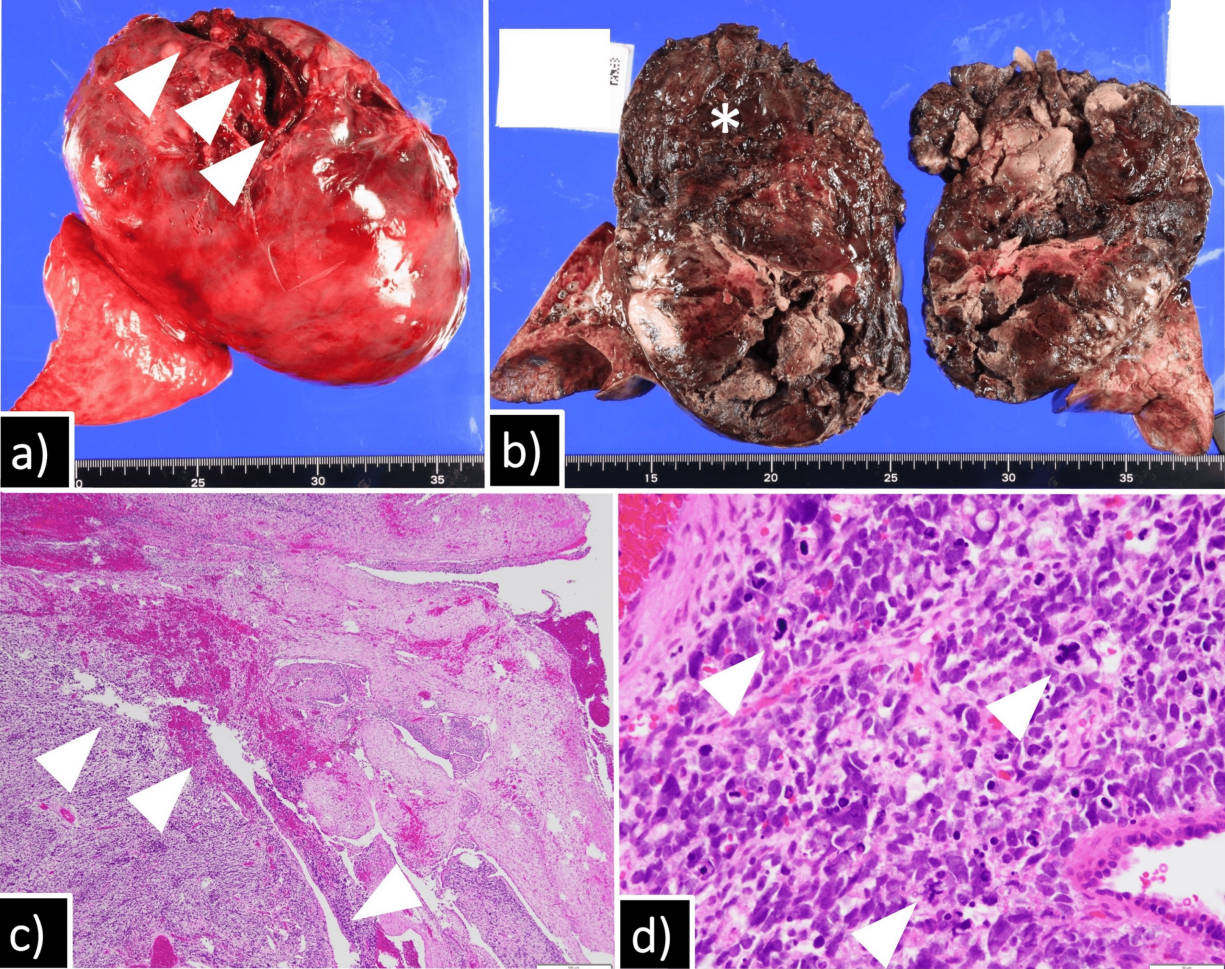
Macroscopic and microscopic findings of the tumor Grossly, the tumor was covered by a capsule with focal disruption (arrowheads) (a). On the cut surface, the tumor (asterisk) was attached to the visceral pleura. The cut surface was dark red due to intratumoral hemorrhage (b). Microscopic examination showed polymorphic atypical tumor cells with multiple cystic components (arrowheads) (c). High-power magnification demonstrated tumor cells with a high nuclear-to-cytoplasmic ratio and atypical mitotic figures (arrowheads) (d).

Macroscopically, the tumor was covered with a capsule with some disruption. The cut surface was dark red, consistent with intratumoral hemorrhage. Microscopically, the tumor consisted of polymorphic atypical cells with a large nuclear-to-cytoplasmic ratio and multiple mitotic figures, along with cystic components, consistent with pleuropulmonary blastoma type II (Figure 3a-3d).

The postoperative course was uneventful. The patient was discharged from the intensive care unit on postoperative day 8 and transferred to the oncology service on postoperative day 11. She completed nine courses of chemotherapy consisting of vincristine, actinomycin-D, ifosfamide, and doxorubicin. At eight months postoperatively, she remains disease-free. Genetic testing, performed because of her current diagnosis and prior hamartomas, identified a known pathogenic germline variant in the DICER1 gene (NM_177438), c.4407-4410del (p.Ser1470Leufs*19), confirming the diagnosis of DICER1 syndrome (MIM: 606241).

## Discussion

We report a rare case of pleuropulmonary blastoma presenting as an OE. Oncologic emergency is defined as a life-threatening condition directly related to a tumor or its treatment [1]. The underlying mechanisms can be broadly classified into three categories: mass effect, vascular or hematologic abnormalities, and metabolic disturbances [2]. The clinical presentation depends largely on tumor location and the structures involved. In the thoracic region, possible manifestations include pneumothorax, hemothorax, cardiac tamponade, airway obstruction, and hemodynamic instability [7]. Up to 16% of cases with these presentations potentially result in death [8]. Anterior mediastinal tumors are one of the most common etiologies and include lymphoma, leukemia, or solid malignancies such as neuroblastoma, germ cell tumor, rhabdomyosarcoma, and Ewing sarcoma [9]. At the same time, OE due to a large pleuropulmonary blastoma is extremely rare but has been reported [3]. PPB is an extremely rare pulmonary malignancy, accounting for only 0.25%-0.5% of all primary lung tumors, yet it is the most common primary lung malignancy in childhood [5]. Approximately 60% of patients are symptomatic at presentation, with clinical manifestations ranging from mild respiratory symptoms to severe cardiopulmonary compromise [6].

The respiratory failure observed in the current case was most likely caused by increased airway resistance due to tracheal deviation and complete right lung atelectasis, which were elicited by the direct mass effect of the tumor. In addition, anesthesia and sedation for intubation exacerbated airway collapse through the loss of thoracic and abdominal muscle tone, which was crucial for maintaining airway patency [10]. Indeed, it is well documented that the risk of anesthesia is extremely high in patients with large anterior mediastinal masses [11]. In the present case, the tumor was located in the thoracic cavity rather than the mediastinum; however, it caused tracheal deviation and complete collapse of the right lung. Therefore, a similar anesthetic-related event was anticipated, and percutaneous biopsy under minimal sedation and local anesthesia was considered. Nonetheless, the patient's agitation precluded this approach. Intubation and management of cardiopulmonary conditions under anesthesia were performed with extreme caution and were initially successful. However, the patient gradually required high PEEP to maintain airway patency. Overall, pulmonary decompensation in the present case was likely due to loss of thoracic and abdominal muscle tone required for airway patency by anesthesia, in combination with the direct mass effect of the tumor on the right lung and the trachea. Along with pulmonary decompensation, systemic circulatory insufficiency occurred simultaneously, requiring multiple fluid boluses to maintain adequate blood pressure. This was likely caused by obstruction of systemic venous return due to the large intrathoracic tumor occupying the entire right chest cavity, leading to decreased cardiac preload and subsequent obstructive shock [12]. Furthermore, high PEEP applied for airway management increased intrathoracic pressure and further impaired venous return, contributing to worsening ventilation/perfusion mismatch [13].

After the onset of obstructive shock, the most important intervention is removal of the underlying pathology causing venous flow obstruction; therefore, in the present case, emergency surgical decompression was imperative [12]. Other potential strategies to prevent further deterioration include pre-emptive steroid administration and urgent whole-chest irradiation [13]. However, because the patient was already in a state of shock, these options were not feasible. Furthermore, macroscopic examination of the resected tumor revealed a large volume of intratumor hemorrhage, suggesting that steroid administration or urgent whole-chest irradiation would have been ineffective for preoperative tumor volume reduction.

The hemi-clamshell thoracotomy provided an excellent surgical field and allowed rapid access to the entire tumor in the present case. The approach has been reported to provide excellent surgical exposure and entire resection of the tumor in the cervicothoracic junction or mediastinum while preserving nearby nerves and vascular structures in pediatric patients [14]. Given the preoperative cardiopulmonary compromise in the present case, the first-line objective of the thoracotomy was to exteriorize the tumor as quickly as possible to decrease the pressure in the chest for restoration of adequate cardiopulmonary status. From this perspective, the approach enabled surgeons to achieve a wider opening in the chest than that provided by a conventional posterolateral thoracotomy, thereby facilitating easier exteriorization of the tumor. Another benefit of this approach was access to the hilar vessels and bronchial tree. Posterolateral thoracotomy is not ideal for exposing these structures, particularly if the tumor is large enough to occupy most of the chest cavity [15]. On the other hand, the hemi-clamshell approach can provide views of the hilum, mediastinum, and diaphragm from various angles, and this advantage can be maximally taken into account if major bleeding occurs from large vessels around the mediastinum or hilum [16]. In the current emergency setting, the hemi-clamshell approach allowed prompt tumor exteriorization, restoration of cardiopulmonary function, and safe oncologic resection, resulting in excellent recovery [17]. From a viewpoint of morbidity related to the approach, Christison-Lagay et al. reported excellent functional recovery without long-term evidence of weakness, neuropraxia, or vascular compromise in their 17 cases of "trap-door" or "clamshell" thoracotomy for cervicothoracic malignancies [14]. Kuroda et al. also reported an uneventful recovery following this approach in their case of a neonatal tumor [18]. In the present case, no morbidity has been observed; however, close observation for late-onset muscle weakness, neuralgia, chest wall deformity, and limitation of joint movement is ongoing.

She underwent genetic testing and was diagnosed with DICER1 syndrome postoperatively for a history of hamartoma resection during infancy. Genetic testing for DICER1 mutation is strongly recommended for all cases of PPB, as approximately two-thirds of patients have the pathogenic variant and are at high risk of developing other DICER1-related diseases [19]. In addition, the analysis is recommended for infants presenting with pulmonary cysts [20]. Identification of a known pathogenic DICER1 mutation suggests the cystic lesion could be type I PPB, and pre-emptive resection should be considered before it progresses to the more aggressive type II or III PPB.

In the present case, the short-term outcome is favorable, with no signs of recurrence; however, the patient should remain under close observation, as the reported cure rates after multimodality treatment for type II and type III PPB are approximately 70% and 50%, respectively [19].

## Conclusions

In conclusion, we report a rare case of pleuropulmonary blastoma presenting as a thoracic OE. The patient required sedation and intubation for diagnostic workup; however, her cardiopulmonary function subsequently deteriorated, necessitating an emergent thoracotomy for decompression of the chest cavity and mediastinum. Early recognition of the severity of the condition and prompt surgical intervention were essential. The hemi-clamshell thoracotomy proved to be an effective surgical approach in this critical situation, as it enabled prompt exteriorization of the tumor and decompression of the chest cavity and mediastinum, thereby restoring cardiopulmonary function.
